# Pregnancy Loss Following Amniocentesis or CVS Sampling—Time for a Reassessment of Risk

**DOI:** 10.3390/jcm3030741

**Published:** 2014-07-08

**Authors:** Caroline Ogilvie, Ranjit Akolekar

**Affiliations:** 1 Guy’s and St. Thomas’ NHS Foundation Trust and King’s College, London SE1 9RT, UK; 2 Medway Maritime Hospital, Gillingham, Kent ME7 5NY, UK; E-Mail: ranjit.akolekar@nhs.net; 3 King’s College Hospital, Denmark Hill, London SE5 9RS, UK

**Keywords:** miscarriage, procedure-related pregnancy loss, amniocentesis, chorionic villus sampling (CVS), non-invasive prenatal testing, cell free fetal DNA

## Abstract

Risk of procedure-related pregnancy loss is currently widely quoted in the UK as 1% for amniocentesis and 1.5% for chorionic villus sampling. Published data suggest that these risk figures are out of date and inaccurate, and that new guidelines are required for pre-test counseling. It is our opinion that accurate and evidence-based information concerning miscarriage risk is vital when counseling women, as exaggeration of this risk may deter women from testing, or cause unjustified remorse if a miscarriage ensues. It is also essential that health-care economists are aware of the up-to-date evidence on “procedure-related risk” when applying risk-benefit analysis to assess new technology for non-invasive screening.

## 1. Introduction

When prenatal screening for trisomy 21 (Down syndrome) was first introduced, it was at a time when the cost of invasive sampling, followed by culture and karyotyping of the samples, was substantial, and invasive testing for the whole pregnant population was therefore unaffordable in a state-funded health service. In addition, there was a perceived risk of pregnancy loss following invasive testing. However, the cost of diagnostic testing had to be weighed against the life-time costs of caring for a handicapped child. Prenatal screening was introduced in an attempt to balance these costs. Screening methodologies such as the integrated and combined tests [1,2,3,4] select for invasive testing those women who have the highest risk of trisomy 21, thus reducing the cost of population invasive testing whilst detecting a high proportion of trisomy 21 pregnancies. In the UK, women whose risk of trisomy 21 is higher than a certain threshold (currently 1 in 150) can opt for state-funded invasive testing for confirmation or exclusion of Down’s syndrome; this threshold is chosen by the National Screening Committee, which takes into account the economics of sampling and testing pregnancies and which also considers the risk of miscarriage associated with the invasive procedure itself (a risk-benefit analysis).

Since prenatal screening was introduced, the cost of diagnostic testing has fallen dramatically, due to the replacement of culture and karyotyping of prenatal samples with rapid and accurate molecular testing [5,6]. Additional information regarding the status of the fetus can also be obtained from array comparative genomic hybridization (CGH) testing of material obtained from invasive testing, although most centres currently restrict these tests to pregnancies where there is evidence from ultrasonography of fetal structural anomalies. Importantly, the factors influencing the risks of invasive testing have also changed since the original studies used to determine these risks were carried out. The technology has improved, ultrasound machines have better resolution, operator experience has increased, and cell-free DNA (cfDNA) testing is now available. It is high time that we review these procedure-related risks so that women are provided with accurate estimation of risks rather than historical figures.

## 2. Discussion

The landmark publication by Tabor *et al.* in 1986 [7], describing the results of a randomised control trial of pregnancy loss following amniocentesis, led to a recommendation by the UK Royal College of Obstetrics and Gynecology (RCOG) Guidelines that women should be counseled that the risk of miscarriage following amniocentesis is 1%. There have been no randomized studies comparing women undergoing chorionic villus sampling (CVS) with those having no procedure; the risk for CVS has therefore been approximated from studies comparing amniocentesis with CVS, leading the RCOG guidance to state a procedure-related risk of 1.5% [8,9,10]. The risk figure for pregnancy loss following amniocentesis that has long been quoted in North America, based on expert opinion, is 0.5% [11]. However, a number of publications since the Tabor study have called into question these risk figures and it is generally acknowledged that improvements in technology and increase in operator proficiency are likely substantially to change these risks; nevertheless, these are still the figures used in counseling pregnant women in most genetics clinics and fetal medicine units in the UK, and recent evidence on this topic is largely ignored. A key publication is that of Akolekar *et al.* [12], where data from more than 30,000 pregnancies was used to address the issue of adjusting for maternal demographic characteristics and components of first-trimester screening between the invasive and non-invasive groups prior to assessment of procedure-related loss. This study examined the risk factors for stillbirth and miscarriage at 11–13 weeks and investigated the contribution of CVS towards the final risk. The authors point out that the calculation of procedure-related risk of pregnancy loss by comparison of pregnancy outcome in women undergoing CVS, compared with those that do not have an invasive test, is likely to overestimate the procedure-related risk. This is because the same components of combined screening that lead to an increased risk for fetal aneuploidies and therefore uptake of CVS, such as an increased fetal nuchal translucency and decreased maternal serum pregnancy associated plasma protein-A (PAPP-A), are also associated with an increased risk for miscarriage and stillbirth. Therefore, in studies estimating procedure-related risk following prenatal sampling, it is important to adjust for maternal and pregnancy characteristics before comparing those that had an invasive test with those that did not. The authors report that after adjustment for such characteristics in their study and in control groups, using multivariate logistic regression, the addition of CVS to the model was not statistically significant. This complexity in risk assessment is also highlighted in the Society of Obstetricians and Gynaecologists of Canada Committee opinion on mid-trimester amniocentesis fetal loss rate [11], which states that many variables such as parental age, previous obstetric history, medical history, and timing and methodology of screening for aneuploidy influence, an individual’s background risk of pregnancy loss. Therefore, there is no single percentage that can be quoted as the risk for procedure-related loss, as miscarriage risk is unique to the individual and is based on multiple variables. A recent review of evidence on procedure-related loss demonstrates that the evidence base is clearly conflicting, but points out that the majority of the studies that examine the rates of pregnancy loss in those women who have an invasive procedure and those that don’t, report no significant difference between the groups; the review concludes that the risk of pregnancy loss is unique for each patient and depends on maternal demographic characteristics and components of first-trimester screening test for that individual patient, and that invasive sampling is unlikely to contribute significantly to the risk [13]. 

The advent of aneuploidy screening using cell free fetal DNA (cfDNA) in maternal blood is now creating a new prenatal revolution. This screening methodology is highly accurate, with detection rates for trisomy 21 around 99% [14]. However, the results are not diagnostic, and can take up to two weeks to reach the patient, ruling out first trimester terminations for many women. In addition, this screening is currently expensive compared with the low-cost screening of metabolites in maternal blood, such as in the combined and integrated screens, and therefore unaffordable for a state-funded health service, at least for the entire population. Different models for clinical implementation of cfDNA technology are being considered and in the near future, there will a cost-effective way of making this available to the wider population; however, until such a time, the decision for testing should be based on accurate information rather than fear of pregnancy loss, as a recent qualitative study suggests [15]. Accurate estimates of the risk are therefore essential in order to inform the future direction of prenatal diagnosis, and to give women realistic information on the choices they face. Therefore, whatever the reason for a proposed invasive test, whether a high risk on the combined, quad, integrated or NIPT screen, or an abnormal ultrasound scan, the woman’s decision as to whether to proceed with the invasive test should be based on accurate information.

During pregnancy, women feel at their most vulnerable; delight, anticipation and excitement have a dark underside of anxiety and fear. Many feel that the healthcare process strips them of their individuality and dignity, and they are presented with facts and figures which they must take on trust, unless they are fortunate enough to have the background and resources to question and research the primary data. Good professional practice should dictate that women are counseled using accurate, up-to-date information. The impact of such a change in counseling might, in theory, result in an increase in the uptake of invasive testing, with the associated financial implications. However, the availability of rapid and cheap standalone testing for Down’s syndrome (and other trisomies) [6,16] means that this approach would increase the detection of Down’s syndrome by testing more at-risk women, and for the time being would remain a cheaper option than the sophisticated cfDNA technologies currently being introduced for highly accurate noninvasive screening [17]. Indeed, a review of the whole field of prenatal screening and diagnosis concludes that large-scale prospective trials of these new noninvasive technologies will be required before they can replace existing approaches, and, given the low risk of procedure-related pregnancy loss and the less than 100% detection rate of noninvasive screens, women should have access to invasive testing on demand for conclusive diagnostic information on aneuploidy [18]. 

Inaccurate figures for the risk of miscarriage following invasive prenatal sampling have wide-reaching implications: women may avoid invasive testing, resulting in an affected pregnancy proceeding to term, and women who choose to have an invasive procedure are likely to suffer from unnecessary guilt and remorse if their pregnancy subsequently miscarries. Accurate and patient- and operator-specific information, together with properly informed counseling are essential for ethical and responsible antenatal care. We would recommend regular audits and surveillance of those undertaking CVS or amniocentesis, to assess individual operator competence; these figures should be reported to a national database for monitoring purposes and should be available to women who are considering invasive testing. In addition, women should be informed of the average post-procedure loss rate at the centre which they attend. Best practice guidelines should be revised to recommend a maximum level of post-procedure pregnancy loss (calculated against the background loss) for any individual operative, Inaccurate generic figures when used to calculate risk-benefit for determining national policy on the availability of invasive prenatal testing, will be restricting justifiable access to prenatal diagnosis. A change in current practice is long overdue.

## 3. Conclusions

Generic figures for the risk of pregnancy loss following amniocentesis or chorionic villous sampling should no longer be used. Average operator-specific risks are more appropriate, and women should be counseled to understand that miscarriage risk following an invasive procedure is very low, and that any pregnancy loss is likely to be due to other pregnancy-related and maternal factors.
